# A Step Closer to the “Fourth 90”: A Practical Narrative Review of Diagnosis and Management of Nutritional Issues of People Living with HIV

**DOI:** 10.3390/diagnostics11112047

**Published:** 2021-11-04

**Authors:** Davide Fiore Bavaro, Paola Laghetti, Mariacristina Poliseno, Nicolò De Gennaro, Francesco Di Gennaro, Annalisa Saracino

**Affiliations:** 1 Clinic of Infectious Diseases, University Hospital Policlinico, University of Bari, 70124 Bari, Italy; paola.laghetti1991@libero.it (P.L.); nico84degennaro@gmail.com (N.D.G.); cicciodigennaro@yahoo.it (F.D.G.); annalisa.saracino@uniba.it (A.S.); 2 Unit of Infectious Diseases, Policlinico “Riuniti”, 710121 Foggia, Italy; polisenomc@gmail.com

**Keywords:** HIV, diabetes, cardiovascular disease, chronic kidney disease, liver disease, NASH, NAFLD, diagnosis, nutrition, nutrition management, PLWH

## Abstract

The quality of life of people living with HIV (PLWH) has remarkably increased thanks to the introduction of combined antiretroviral therapy. Still, PLWH are exposed to an increased risk of cardiovascular diseases, diabetes, chronic kidney disease, and liver disease. Hence, the purpose of this review is to summarize the current knowledge about diagnosis and nutritional management with specific indication of macro and micronutrients intake for the main comorbidities of PLWH. In fact, a prompt diagnosis and management of lifestyle behaviors are fundamental steps to reach the “fourth 90”. To achieve an early diagnosis of these comorbidities, clinicians have at their disposal algorithms such as the Framingham Score to assess cardiovascular risk; transient elastography and liver biopsy to detect NAFLD and NASH; and markers such as the oral glucose tolerance test and GFR to identify glucose impairment and renal failure, respectively. Furthermore, maintenance of ideal body weight is the goal for reducing cardiovascular risk and to improve diabetes, steatosis and fibrosis; while Mediterranean and low-carbohydrate diets are the dietetic approaches proposed for cardioprotective effects and for glycemic control, respectively. Conversely, diet management of chronic kidney disease requires different nutritional assessment, especially regarding protein intake, according to disease stage and eventually concomitant diabetes.

## 1. Introduction

The introduction of combined antiretroviral therapy (cART) has remarkably reduced the morbidity and mortality of people living with HIV (PLWH) in recent decades by reducing AIDS-related complications, thus substantially improving their quality of life. The Joint United Nations Programme on HIV/AIDS proposes that by 2020 and 2030, at least 90% of all PLWH in a country should be diagnosed, at least 90% of them should be on ART and at least 90% of those on ART should be virologically suppressed [1]. At the same time, it has become clear that controlled viral load cannot be the ultimate goal in PLWH care, as 90% of them should report a good health-related quality of life, defining a new “fourth 90” [2,3]. However, the achievement of the “fourth 90” is far from being reached, and further attempts should be made by clinicians and healthcare systems to obtain this target, with an even stronger effort addressed to “fragile” populations [4].

HIV-related chronic inflammation, associated with progressive glucose and lipid dysfunction [5], unhealthy lifestyles (inappropriate diet, low physical activity, smoking, and drug use), and age-related noncommunicable diseases due to increased life-expectancy [6] importantly enhance the risk of premature cardiovascular diseases, type 2 diabetes, non-AIDS-related cancers, and end-stage organ diseases for PLWH [7].

Moreover, although the metabolic impact of antiretroviral medications has been remarkably reduced by the diffusion of highly tolerable and effective modern cART regimens, these still have to be considered as lifelong therapies, with a toxicity burden that should be taken into account in the long term.

Given the exposure of PLWH to metabolic comorbidities, their early diagnosis, the assessment of risk factors, and correct nutrition deserve a central role in the management of chronic HIV infection and its related metabolic complications, since it directly affects body weight, lipid profile, blood pressure, glucose metabolism, oxidative stress, and inflammation and, consequently, impacts on the risk of cardiovascular diseases, diabetes, and obesity development [8,9,10,11,12].

Accordingly, the importance of nutritional intervention was exhibited by a 2019 clinical controlled trial showing how dietary modifications can improve oxidative stress associated with metabolic and chronic comorbidities (obesity, hypertension, diabetes, and dyslipidemia), by increasing the consumption of food containing antioxidant molecules such as polyphenols, by reducing the adipose tissue and improving the gut microbiome [13].

In this setting, the recent COVID-19 pandemic has contributed to further worsening lifestyle habits both in PLWH and in the general population, as sedentary, low physical activity, and unhealthy eating behaviors became common in the course of “locking-down” emergency procedures enforced by different countries [14,15]. Additionally, race and economic disparities limiting access to HIV care have dramatically grown during the last two years, reducing or stopping, usual outpatient and inpatient services, access to treatments, medical appointments, as well as investigations for screening or follow up of metabolic diseases [16,17].

Consequently, an emerging metabolic diseases epidemic could be hidden around the corner [18] if proper measures are not quickly undertaken—particularly in PLWH, who are, accordingly to the abovementioned reasons, overexposed to these complications. In this regard, an early diagnosis of HIV comorbidities is pivotal to a successful global management of PLWH.

Hence, the purpose of this review is to summarize the current knowledge about prompt diagnosis of comorbidities in PLWH and the consequent related nutritional management, taking into account metabolic dysfunctions and cART metabolic toxicity, and ultimately providing practical dietary indications to physicians and nutrition specialists.

## 2. Nutritional Suggestions According to Different Comorbidities

### 2.1. Cardiovascular Diseases

Cardiovascular diseases (CVD) have an important impact on PLWH, since they significantly increase their risk of mortality compared to the general population; on the other side, HIV infection itself is an independent risk factor for cardiovascular diseases, especially coronary artery disease and chronic heart failure [19,20]. Alonso, A. et al. [21] demonstrated, through the analysis of a large insurance database including around 20,000 PLWH, an elevated risk of chronic heart failure and myocardial infarction in this population, independently of other risk factors, underlining the importance of adopting preventive measures for CVD in PLWH [17]. This increased risk could be explained by multiple factors. At first, the predominant role of HIV infection is demonstrated by the strong correlation between the T CD4+ cells count nadir, persistent HIV replication, and the risk of CVD development [22,23]. Indeed, the continuous viral replication within lymphoid tissue perpetuates the existence of a pro-inflammatory state [24,25,26] by upregulating the inflammatory cytokines, such as C-reactive protein, high sensitivity C-reactive protein, IL-6, and D-dimer; deregulating the CD8+ activity; and activating monocytes and macrophages. This process, in turn, enhances the atheromatic plaque formation, arterial thickness and stiffness, and endothelial alteration, and promotes cardiovascular events [27,28,29]. Additionally, PLWH have a higher-than-average plasma level of oxidized low-density lipoprotein (oxLDL) and activated monocytes with oxLDL receptors. The oxLDL are the inflammatory form of LDL, found in atheromatous plaque that contribute to foam cells formation [30]. Moreover, HIV itself could modulate the levels of fatty acids synthase, causing alterations of plasma lipid profile [31]. Not least, the chronic inflammation is also responsible for epigenetic alteration such as DNA methylation and upregulation of the systemic inflammatory markers, microRNAs [32,33].

Secondly, a key role in the development of the pro-inflammatory environment is played by gut bacterial translocation and altered immune response. It is demonstrated that even after a long period of cART, the depression of gut-associated lymphoid tissue that occurs during HIV acute infection, does not recover entirely [34,35,36]. This is responsible for alteration of gut mucosal integrity, facilitating the translocation of microbes, their related products (such as lipopolysaccharide), and other intestinal antigens such as food antigens, which constantly stimulate and hyperactivate the immune system [37,38,39,40].

The extent to which antiretroviral medications are involved in enhancing the incidence of CVD in PLWH is historically a matter of debate that has led to a deep investigation of the potential cardiovascular toxicity of each class of antiretroviral drugs. Among nucleoside reverse transcriptase inhibitors (NRTIs), abacavir was traditionally related to an increased risk of cardiovascular disease in HIV-positive patients, which has been associated with mechanisms of endothelial disfunction and imbalance in platelets’ activation and aggregation processes [41]. Although the impact on the cardiovascular system of exposure to abacavir remains controversial, several alternative therapeutic strategies are currently available to avoid this risk, above all opting for agents of the same class with different toxicity profiles, such as combination regimens based on tenofovir disoproxile fumarate (TDF) rather than tenofovir alafenamide (TAF) [42,43]. In recent years, the advent of highly efficient antiretroviral drug classes such as protease inhibitors (PIs) and, lately, integrase strand transfer inhibitors (INSTIs), allowed the creation of combined antiretroviral regimens that avoided NTRI-related cardiovascular toxicity along with maintaining high control on viral replication also in experienced subjects. It should be noticed that these drug classes have also been associated with increased total cholesterol and triglycerides levels and decreased high density lipoproteins (HDL) levels, with a resulting pro-atherogenic lipid profile and, along with non-nucleoside reverse transcriptase inhibitors (NNRTI), with dyslipidemia and lipodystrophy [44].

The role of cART in dyslipidemia is still not fully clear but could be partially explained by alteration of triglycerides hepatic synthesis and alterations of retinoic acid and LDL-binding proteins [45,46]. In any case, recently developed antiretroviral drugs, such as raltegravir, dolutegravir, bictegravir, rilpivirine and doravirine have a safer impact on lipid profile compared to older ones (such as zidovudine, stavudine, nevirapine, and partially lamivudine); two-drug combination regimens are also a modern therapeutic option that could permit, importantly, in selected PLWH, decrease of the pharmacological burden without losing efficacy in virological suppression [47,48].

To assess the CVD risk and to facilitate an early diagnosis of CVD, different algorithms considering different factors (such as smoking habits, lipid profiles, comorbidities, waist circumference, body mass index, blood pressure) have been developed. The most common used algorithms are the Framingham Score, which is more accurate if used for the US population, and the Systematic Coronary Risk Evaluation tool, developed by the European Society of Cardiology, in turn more suitable for the general population [49]. PLWH, along with traditional CVD risk factors, have the exposure to cART as an additional risk factor. Hence, a specific algorithm for this population has been designed by the Data Collection on Adverse Events of Anti-HIV Drugs (DAD) group [44]. In this regard, the European AIDS Clinical Society guidelines [50], suggests the use of the Framingham equation or other system recommended by local national guidance and to repeat these assessments annually, in order to start any needed intervention in time.

After an appropriate diagnosis, in order to reduce the risk of further deterioration in this population, a great impetus should be given to modifiable risk factors, such as healthier lifestyles, smoking interruption and, more importantly, healthier eating behavior.

Different dietary approaches (Table 1) have been proposed to reduce cardiovascular risk and were discussed in depth in a recent review of Rychter et al. [51], although data on PLWH are very scarce. The 2019 ACC/AHA Guideline on the Primary Prevention of Cardiovascular Disease recommends a plant-based or Mediterranean diet as cardioprotective diets [52]. The latter, based predominantly on vegetables, legumes, nuts, fruits, extra-virgin olive oil, whole cereals, and fish consumption and a moderate intake of dairy products and red meats, is associated with almost 30% reduction risk of myocardial infarction, stroke, and cardiovascular mortality, as observed during the PREDIMED randomized trial [53]. Furthermore, the Mediterranean diet seems to have a positive effect not only in primary but also in secondary prevention of myocardial infarction, specifically. A prospective analysis, carried out on 553 patients, demonstrated a positive correlation between adherence to this diet and successful myocardial reperfusion [54] Another dietary approach to reduce CVD incidence, and, in particular, hypertension, is the DASH (dietary approach to stop hypertension) diet. The DASH diet focuses primarily on fruit, vegetables, fat-free/low-fat dairy, whole grains, nuts, and legumes intake, and, differently from the Mediterranean diet, it demands a minor use of extra-virgin olive oil. This diet is associated with a reduction in both systolic and diastolic blood pressure, total cholesterol, and LDL but did not show any impact on HDL levels and triglycerides [55,56]. Vegetarian diets can also be implemented due to their cardioprotective effect. This diet has lower energy density, total cholesterol, LDL, and HDL than the “omnivorous” diet, and showed an important effect in terms of reduction of blood pressure, a lower rate of mortality by stroke in men (but no significant difference in women) [57], and an inverse correlation with cardiovascular endpoints [58]. Finally, the portfolio dietary pattern is another diet style associated with a 10-year reduction of coronary heart disease risk [56], which focuses on consumption of “cholesterol-lowering food” such as nuts, apples, oranges, berries, soluble fibers from oats, barley, psyllium, okra or eggplant, and vegetal protein from legumes or soy products [59,60,61]. In addition, this nutrition regimen focuses on dietary integration with 2 g plant sterols provided in a plant-sterol-enriched margarine. In particular, atherosclerosis, characterized by the progressive accumulation of inflammatory cells and lipids within the arteries’ intima, benefits from the consumption of specific nutrients and foods, such as omega 3 fatty acids, polyphenols, plant sterols, fibers, olive oil, lycopene, vitamins (C, E, B), and soy, as reviewed in the work of Torres and colleagues [62]. This can be explained by the anti-inflammatory and antioxidant properties of these compounds, which are able to positively affect hypertension, to prevent LDL oxidation, leucocyte migration, and production of adhesins and chemokines [62].

Regardless of the type of nutritional approach, reduction of body weight and abdominal fat is a pivotal point in the improvement of cardiovascular risk. In any case, a hypocaloric diet should be recommended to overweight patients and a body mass index (BMI) of 20–25 kg/m^2^ and a waist circumference <94 cm and <80 cm for men and women, respectively, are desirable targets.

Overall, calories resulting from total fat intake should be about 30% of total calories and those resulting from carbohydrates intake should be between 45% and 55%. Concerning micronutrients intake, guidelines [52] suggest to not exceed 5 g per day of sodium intake, to avoid trans fatty acids, and to consume less than 10% of calories resulting from saturated fat and less than 300 mg per day of cholesterol. Eggs, because of their high content of cholesterol, are always controversially associated with CVD risk. In fact, a 2016 meta-analysis demonstrated no association between egg intake and CVD risk and a reduced risk of heart stroke of 12% with a higher consumption of eggs [63]. Therefore, egg consumption could be allowed in the context of a varied and balanced diet. Finally, due to its hypocholesterolemic effect, a quantity of 25–40 g of fiber should be consumed daily [52].

In addition, another condition linked to inflammation, thrombosis, endothelial alterations, and HIV infections, which can itself benefit from a nutritional intervention, is pulmonary hypertension. In particular, vitamin D, vitamin C, and iron deficiency have been observed in patients with this disease, while a diet rich in flavonoids, isoflavones, and resveratrol is associated with a slower progression of this disorder in animal models [64].

However, to the best of our knowledge, few studies investigated the improvement in cardiovascular risk in PLWH populations submitted to a dietary intervention. A randomized controlled trial by Stradling et al. [65], investigating a population of adult PLWH on cART and with high levels of LDL, showed that a Mediterranean diet enriched with hypocholesterolemic food was able to adequately control blood pression and reduce by 10% the levels of LDL-cholesterol, if compared to diets focused on the reduction of saturated fat alone [52].

### 2.2. Type II Diabetes

The incidence of Type 2 diabetes (T2D) is remarkable in PLWH and even higher than that observed in general population [66]. In this sense, an early T2D diagnosis is crucial to prevent related complications. According to EACS Guidelines [50], to assess a pre-diabetic condition, fasting glucose has to be evaluated in all newly diagnosed PLWH and all PLWH about to start cART. Altered findings should be repeated and if the test is indicatory for glucose metabolism alteration (fasting plasma glucose 5.7–6.9 mg/dL), an oral glucose tolerance test measurement is recommended. In particular, a 2018 work of Celho and colleagues highlights the relevance of the oral glucose tolerance test as an earlier diagnostic marker of glucose impairment, compared to glycosylated hemoglobin, which seems to underestimate glycemia levels in PLWH compared to the general population [67]. Accordingly, EACS Guidelines point out that glycosylated hemoglobin tends to underestimate diabetes in PLWH under antiretroviral treatment, in particular those under abacavir. Furthermore, the guidelines do not recommend glycosylated hemoglobin in cases of hemoglobinopathies, increased erythrocyte turnover, and severe liver or kidney dysfunction, as well as in cases of supplementation with iron, vitamin C, and E, and with a patient age > 70 because of unreliable values [50]

HIV-related chronic inflammatory processes, despite the beginning of antiretroviral treatments, are probably the major factor responsible for this phenomenon, independently of antiretroviral medications and noncommunicable comorbidities [68]. In this regard, a case control study of Brown and colleagues on 55 subjects matched on baseline BMI and race/ethnicity, showed that persistently (within the first 48 week after cART initiation) high serum levels of inflammation markers independently predicted T2D development [68].

Additionally, low T CD4+ cells count, high viral load, and HCV coinfection are all independently associated with the risk of developing mechanisms of insulin resistance, altered fasting glucose levels, and, consequently, T2D. Notably, a lower T CD4+ cells nadir has been associated with impaired glucose tolerance and a higher risk of diabetes [69,70]. In this sense, the importance of a rapid virological suppression is important for reducing the risk of diabetes, along with the reducing risk of producing drug-resistant strains in case of virological failure [71].

Chronic antiretroviral drugs consumption could further promote T2D occurrence in PLWH, especially in the case of prescription of first-generation PIs (indinavir, lopinavir/ritonavir) and thymidine analogues (stavudine), for which a notable association with insulin resistance was demonstrated [72,73]. In particular, PI-linked diabetes pathogenesis is explainable by the inhibition of glucose transporter GLUT4 [74], which causes peripheral insulin resistance, hyperglucagonemia, and progressive BMI increase, which are responsible, in turn, for emerging insulin resistance [75] and systemic inflammation [68,76].

The role of newer antiretroviral drug classes, such as INSTIs, in the pathogenesis of T2D in HIV positive patients has been deeply investigated. Bictegravir, the latest licensed drug in the INSTIs class, has been recognized as having a potential role in glycemic control impairment [77]. Moreover, the INSTIs drug class as a whole is gaining growing attention for its role in causing weight gain and metabolic syndrome in PLWH, both phenomena possibly leading to an outbreak of insulin resistance and T2D in this special population [78]. It is worthy of mention that no negative effect has been observed for INSTIs on insulin resistance when in NRTI-sparing, dual regimen formulations; on the contrary, insulin resistance was improved in patients undergoing cART simplification for at least 48 weeks [79]. Unfortunately, for all mentioned examples, literature evidence is inconsistent and further studies are required.

Excluding the risk deriving from cART and HIV infection itself, T2D incidence is increased by many preventable lifestyle behaviors: in fact, unhealthy diet, lack of physical activity, smoking, being overweight, and having a large waist circumference are notorious risk factors for this disease [80]. In light of the above, a specific dietary approach (Table 2) could play a central role in prevention and management of PLWH with T2D or dysglicemia. Patients with this comorbidity should follow the nutritional guidelines for T2D as recommended by American Diabetes Association’s 2019 Standards of Medical Care in Diabetes. The importance of lifestyle modification to reduce T2D risks and complications is shown in a recent study of Duncan et al. [81]. Specifically, an individualized diet and physical activity plans were performed in a population of PLWH with impaired fasting glucose with the aim to achieve 10 healthy lifestyle goals (i.e., achieve 7% weight loss, decrease saturated fat to <10% total daily energy intake, restrict sodium to <2.5 g daily, and take 10,000 steps per day). This intervention was successful in reducing the risk of type 2 diabetes in this population [81].

First, overweight or obese PLWH with T2D should reach their ideal weight, since weight control is one of the predominant factors influencing insulin resistance and glycemic levels [82]. The management of weight loss usually requires a diet of 1200–1500 kcal/day for women and 1500–1800 kcal/day for men; or, more precisely, a calorie restriction of 500–750 kcal/day compared to total daily energy expenditure [83].

Assuming that all meal plans should be individualized and redacted on personal preferences, metabolic goals, and lifestyle, different studies show that the Mediterranean Diet has effective results on weight loss and glycemic control and should be the diet of choice when possible [84]; in any case, a low-carbohydrate diet should be prescribed to improve glycemia value and reduce the use of hypoglycemic drugs in these subjects. In addition, carbohydrate sources should be rich in fiber, such as whole grains, fruit, and vegetables, and the use of sweetened beverages and food rich in refined sugars should be avoided.

Concerning other macronutrients, an important stratification of dietary needs is based on presence of chronic kidney diseases in the course of T2D. Indeed, PLWH without kidney impairment could consume the same amount of protein as the general population, that is about 1–1.5 g/kg body weight, or even higher according to physical activity and muscular mass [85]. Furthermore, a diet that contemplates a high amount of protein (about 20–30% of total calories) increases the sense of satiety in people with T2D, compensating the reduced quantity of carbohydrates [86]. Conversely, in the case of kidney impairment, and in particular microalbuminuria or reduced glomerular filtrate, the safest amount of protein is not higher than 0.8 g per kg of body weight.

Finally, regarding fats consumption, foods rich in polyunsaturated or monounsaturated fats, such as nuts, seeds, olive oil, and fish (in particular salmon, tuna, anchovy, mackerel, herring, etc.) could contribute to improved glycemic control and blood lipids, and should be included in the dietary plan [85].

Future studies and diets should also consider the importance of the gut microbiome, which is significantly influenced by nutrition, lifestyle, stress, and environment. Particularly, it was demonstrated that T2D and HIV infection both influence the microbiome, lowering the biodiversity of bacterial species. This results in an increase of pro-inflammatory markers and in an alteration of tryptophan metabolism, that is in turn linked to immune activation and increased mortality in PLWH [87]. Accordingly, the use of probiotics in PLWH should be encouraged, since a positive effect has already been demonstrated [88,89] in terms of restoring gut commensal bacteria equilibrium, increasing T CD4+ cells count, and reducing inflammation and immune-activation markers [90,91,92].

In conclusion, in PLWH with T2D, a global nutritional management is crucial to improve glycemic control and to minimize the consequences of T2D in PLWH.

### 2.3. Chronic Kidney Disease

Among non-AIDS related comorbidities, renal function impairment has a prevalence of 30% in PLWH [94]. Traditional risk factors (age, hypertension, diabetes) and HIV-related risk factors (ongoing viremia, impaired immune function, HCV coinfection, and antiviral drugs nephrotoxicity) together with the increased lifespan of PLWH, make renal impairment a major concern in the management of these people [95,96], especially considering that renal impairment predisposes to other comorbidities, including cardiovascular diseases, osteoporosis, etc. [97,98].

Before the introduction of cART, HIV-associated nephropathy was the primary cause of renal disease in PLWH, leading to end-stage renal disease. Nowadays, HIV-associated nephropathy is less frequent, while chronic kidney disease (CKD) is the most common HIV-related renal dysfunction [99]. The Copenhagen Comorbidity in HIV Infection Study [92] has demonstrated that HIV infection is an independent risk factor for renal impairment development, probably because of chronic inflammation and coagulation activation induced by HIV infection. Furthermore, it showed that the risk of renal impairment was higher in older PLWH compared to younger PLWH [100]. In fact, the kidneys act as an HIV reservoir and the resulting polyclonal gammaglobulinemia, immune complex and cryoglobulin production, and glomerular injury are consequences of T-cell-dependent B response hyperactivation. Furthermore, infected macrophages and T-cells secrete proinflammatory cytokines, such as IL-6, causing lasting inflammation and renal injury [101,102]. High values of IL-6 and other immune activation markers are usually detected also in PLWH under cART treatment and with low viral loads [103,104], and HIV itself can downregulate immune regulatory genes, induce apoptosis, and cause accelerated aging [101,105]. All these factors contribute to perpetuate an inflammatory state, leading to CKD and, in some cases to end-stage renal disease.

After the advent of cART regimens, antiretroviral-related renal toxicity has represented a new cause of concern. The association between NRTIs (the first antiretroviral medication available) and kidney injury has been widely described. Mitochondrial dysfunction and tubular cell injury due to the binding of the human mitochondrial DNA polymerase-γ [106], direct renal tubular damage, interstitial nephritis, and crystal-induced obstruction were all included among the possible causative mechanisms potentially explaining the higher prevalence of CKD in PLWH under durable treatment with NRTIs, especially TDF [107]. It should be specified, however, that the association of TDF and CKD is relevant especially after prolonged use, in patients with high baseline risk for CKD, and when associated with a pharmacoenhancer, as boosted-protease inhibitors [108,109,110,111,112]: for these reasons TDF is still recommended as the first-line NA in patients with HBV-decompensated cirrhosis and, in association with emtricitabine, for use as pre-exposure prophylaxis [113].

The development of new antiretroviral medications has come, again, to the aid of clinicians. TAF, a new prodrug of tenofovir, in 2016 has provided a valid alternative to TDF with negligible effect on renal function (and, consequently, on bone density) in a single-tablet-regimen combination with a range of third agents, included boosted-protease inhibitors [42].

In this scenario, an early diagnosis of CKD in PLWH is crucial but often difficult. A review of Chazot and colleagues [114] summarizes the most suitable biomarkers for an early diagnosis and for monitoring the CKD progression in PLWH. In fact, GFR and measurement of proteinuria by the urine protein/creatinine ratio used for CKD diagnosis in the general population, have proven to lack sensitivity in PLWH. For PLWH the best equation to estimate GFR is CKD-EPI study equation, also according to EACS Guidelines [50]. Unlike in the general population, tubular damage represents a large majority of lesions that may affect kidneys in PLWH, and, consequently, low-grade proteinuria is associated with a higher risk of disease progression in this population and can direct clinicians to a more appropriate antiretroviral agent choice. EACS Guidelines [50] suggest using urine dipsticks for screening and define proteinuria as persistent if confirmed on ≥2 occasions >2–3 weeks apart. It is suggested, if the urine dipstick is ≥1+, to check urine albumin/creatinine or protein/creatinine to screen for glomerular disease and both glomerular and tubular disease, respectively. Anyway, urine albumin/creatinine is not suitable to detect tubular proteinuria due to drug nephrotoxicity. In this case, protein/creatinine is more appropriate.

Other biomarkers associated with kidney disease progression and mortality in PLWH are of kidney diseases such as N-acetyl beta glucosaminidase, kdney injury molecule-1, and Alpha-1-microglobulin [114].

Nevertheless, PLWH with (and probably also those without) CKD, of any cause, could take advantages by an accurate dietary plan to counteract and slow down the progression through kidney impairment and, eventually, end-stage renal disease. However, nutritional needs differ depending on the CKD stadium (Table 3).

Overall, it should be noticed that, in subjects with CKD, the resting energy expenditure is higher if compared to non-CKD people because of the inflammatory state and metabolic alterations associated with CKD [115]; moreover, insufficient energy intake could lead to protein catabolism and consequently to a negative nitrogen balance. For these reasons, the calorie intake should be carefully balanced in these subjects to avoid muscle mass reduction and wasting. Consequently, nutritional guidelines suggest a caloric intake between 25 to 35 kcal per kg of body weight [116]. This range should be corrected according to weight status and weight goals, age, gender, level of physical activity, and metabolic stressors. Indeed, CKD patients who consume less than 0.8 g of protein per kg of body weight, with a caloric intake between 15 and 25 kcal per day have a negative nitrogen balance; while when caloric intake from protein is between 25 and 35 kcal per day the nitrogen balance tends to be neutral or positive. This evidence suggested that caloric intake should be higher in patients that do not reach the protein consumption suggested by recommended daily allowance, in order to avoid malnutrition [116].

Moreover, nutritional practice guidelines suggest for nondiabetic and not-on-dialysis patients with glomerular filtration rates (GFR) of 50 mL/minute/1.73 m^2^ or less, a protein daily intake between 0.55 and 0.60 g/kg body weight or a very low-protein diet providing 0.28–0.43 g dietary protein per kg of body weight per day, with additional keto acid/amino acid analogues to meet protein requirements (0.55–0.60 g /kg body weight/day); while for diabetic subjects with CKD it is reasonable to prescribe, under close clinical supervision, a dietary protein intake of 0.6–0.8 g per kg of body weight per day to maintain a stable nutritional status and optimize glycemic control. Patients on maintenance hemodialysis and peritoneal dialysis without diabetes but metabolically stable and with diabetes can consume 1.0–1.2 g/kg body weight of proteins; in those with diabetes also, a higher amount can be considered to improve glycemic control [116]. In fact, it has been demonstrated that a protein-restricted diet with an adequate caloric intake can slow the GFR decline and preserve a good nutritional status [117]. More than half of this protein intake should be based on high-biological-value proteins such as meat, fish, eggs, and dairy products for their content of essential amino acids [118].

To reduce the intake of proteins with low biological value, including cereals and derivatives, and promote the use of foods with high-biological-value proteins, the consumption of “low protein foods” can be discouraged: these industrial foods have very low amount of potassium, sodium, and phosphorus and provide energy without nitrogenous waste products [119,120].

Additionally, in PLWH with CKD, a very important dietary adjustment regards water and electrolyte intake. The consequences of any imbalance could be the development of hypertension, edema, heart failure [121], hyperparathyroidism, calcification of the arteries and heart valves [122], and metabolic acidosis; all resulting in protein catabolism, bone demineralization, insulin resistance, and uremic toxins retention [123].

Accordingly, the 2020 Chronic Kidney Disease Evidence-Based Nutrition Practice Guidelines [116], in order to improve blood pressure control, kidney function, hydration status, reduce the risk of acidosis, suggest:

(i) to adjust potassium (for patients with stage 3–5 of CKD, independently from hyperkalemia) and phosphorus intake in order to maintain serum levels within normal range;
(ii) to adjust sodium intake to <2.3 g per day in subjects with stage 3–5 of CKD;
(iii) a total elemental calcium intake of 800–1000 mg/d (including dietary calcium, calcium supplementation and calcium-based phosphate binders) in adults with CKD 3–4 not taking active vitamin D analogues; and a tailored adjustment for CKD stage 5.

Finally, a proper nutrition, rich in fiber (at least 20–30 g), is fundamental to counteract intestinal microbiota alteration, as already discussed in previous paragraph, that could frequently occur also in the advanced stages of CKD [124]. Once again, guidelines suggest the Mediterranean diet for CKD patients who are not on dialysis to improve their lipid profiles and suggest increasing fruit and vegetables consumption to reduce body weight, blood pressure, and net acid production [116].

In conclusion, improving nutrition in PLWH affected by CKD is pivotal to achieve the maximum state of health and reduce the risk of further kidney impairment, aside from the use of other medications.

### 2.4. Liver Disease

Liver diseases, including fatty liver diseases, viral hepatitis, drug-associated hepatotoxicity, and hepatocellular carcinoma, are possible comorbidities in PLWH affecting their morbidity and mortality.

Notably, HIV has a direct role in liver damage, increasing the risk of cirrhosis in patients with chronic hepatitis viruses infection, alcoholic hepatitis, and non-alcoholic fat liver disease (NAFLD) [125,126,127]. Indeed, HIV viral envelope can interact with hepatocytes enhancing ROS production and oxidative stress; this, in turn, promotes a pro-fibrogenic environment, with activation of the TGFβ1 pathway and secretion of extracellular matrix by stellate cells [128,129]. Development of fibrosis and deposition of extracellular matrix by stellate cells is sustained by Kupffer cells and hepatic macrophages, infected and activated by HIV [130]. In addition, another mechanism involved in liver fibrosis is the translocation of altered gut microbiome in the portal blood and depletion of CD4+ and general immune response deregulation [131].

All these pathogenetic aspects, along with viral hepatitis, contribute to a particularly high incidence of NAFLD in the HIV-infected population. In any case, both in the general population and in PLWH, the primary cause of NAFLD is still obesity and being overweight, independently from other causes [132]; additionally, being overweight and visceral fat increase could be worsened or partially caused by some cART regimens, such as those containing INSTI [133,134], that are currently considered the first-line treatment of HIV infection. Moreover, as demonstrated by Vodkin et al., non-alcoholic steatohepatitis (NASH) is more frequent and severe in HIV patients than in the general population [135].

In this regard, diagnosis of NAFLD should be of primary concern in PLWH, especially in those subjects with metabolic disorders and obesity. Transaminases are not considered a reliable biomarker since they can be found normal in NASH, and, consequently, are not helpful in discriminating between NASH and simple steatosis [136]. Although different biochemical scores have been developed, such as the Nash Test [137], to date, only the magnetic resonance elastography is suitable to this purpose, even before the onset of fibrosis [138]. In addition, transient elastography (Fibroscan, Echosens, Paris) is an easy, rapid and non-invasive tool for the diagnosis of fibrosis (cut-off scores: 7, 8.7, and 10.3 kPa to predict, respectively, fibrosis at least F2, F3, and F4) [139] in association with biochemical scores. Indeed, EACS Guidelines [50] suggest using transient elastography with a controlled attenuation parameter to diagnose HIV-associated NAFLD. Moreover, according to these guidelines, to diagnose NASH, a biopsy showing steatosis, hepatocyte ballooning, and lobular inflammation is necessary.

While it is generally accepted that subjects with compensated liver disease do not have peculiar energy and nutrition requirements, greater attention should be paid to those with advanced liver disease [140]. As pointed out by the 2018 guidance from the American Association for the Study of Liver Diseases [141], in cases of NAFLD or NASH, lifestyle intervention and weight loss are crucial points in the management of these diseases. In fact, a hypocaloric diet allows the mobilization of fatty acids from the liver, and the reduction of at least 3–5% of total bodyweight is sufficient to markedly improve steatosis. A further reduction of 7–10% of total bodyweight could also improve liver fibrosis. Nevertheless, the importance of weight loss is pointed out by the direct correlation between obesity and NAFLD. As a consequence, a rapid weight loss could produce a reduction of up to 60% of liver triglycerides content, reduction of free fatty acids uptake, and improvement of insulin resistance [142]. Following this evidence, the reduction of body weight should be considered the first approach in the treatment of NAFLD and prevention of NASH (Table 4).

However, to date, the more appropriate macronutrients distribution is still debated, but the adherence to a Mediterranean-style diet appears to be able to improve liver steatosis also in the absence of weight loss, compared to the high-fat, low-carbohydrates diet [141]. This can be probably explained by the avoidance in Mediterranean diet of refined and high-sugar foods and the presence of specific aliments (olive oil, fish rich in polyunsaturated fatty acids, nuts, seeds, whole grains, legumes, fruits, and vegetables) known for being beneficial for NAFLD [143].

Apart from the type of diet, also meal frequency and timing could produce an important effect on liver metabolism. For instance, a study of Koopman et al. [144] explored the liver fat accumulation in the course of high-fat, high-sugar and high-sugar hypercaloric diets consumed variously in the principal meal or in multiple meals and snacks during the day. Intriguingly, the habit of snacking during the day, typical of a western diet, was associated with an increase in liver fat, independently of the type of diet [144].

A specific nutrition intervention is needed also in patients with cirrhosis since it can significantly influence morbidity and mortality [145]. In fact, a malnourished state characterizes patients with cirrhosis [146] because of deficient nutrient intake, malabsorption, and altered metabolism of lipids, proteins, and carbohydrates caused by liver dysfunction. Because of portal hypertension and cirrhosis, nutrients are not properly metabolized in the liver and are not fully available for biologic functions [147]. Furthermore, in patients with cirrhosis, it is common to find a deficit of liposoluble vitamins (vitamins A, D, E, and K) because of reduced production of bile salt, highlighting the importance of vitamin supplementation in these subjects [148]. Moreover, cirrhotic liver lacks in glucose synthesis and glycogen storage, and this causes a gluconeogenesis from body protein amino acids even after only one night of fasting, resulting in a muscular mass loss [149]. This is clinically significant, since in these patients only one night of fasting is similar to 2–3 days of starvation in healthy control [150]. Prolonged and constant muscle mass loss leads to sarcopenia, which seems to contribute to hyperammonemia in course of cirrhosis [151]. The impairment of glycogen storage makes it, therefore, necessary to consume multiple meals during the day, in order to avoid hypoglycemia by eating at least 45% to 65% of the total caloric intake as carbohydrates even if the patient suffers from diabetes mellitus [152]. In this regard, the consumption of nutrient-dense snacks before sleeping in the late night seems to increase total body proteins, countering lean mass loss [153].

All these factors explain the pro-catabolic state in patients with cirrhosis and the importance of these aspects in producing a diet plan for these patients. To counteract catabolism, current European guidelines suggest that caloric intake, fixed on patient malnutrition state, should be of 35 to 40 kcal per kg of body weight per day and the protein intake should be of 1.2 g per kg of body weight per day [140]. Interestingly, in the past, it was believed that a protein restriction was necessary to reduce hyperammonemia; however, studies suggested that protein restriction increases muscular protein catabolism— worsening, as a consequence, hepatic encephalopathy and cachexia [154]. Particularly, according to ISHEN (International Society for Hepatic Encephalopathy and Nitrogen Metabolism) guidelines [155], proteins from vegetables and dairy sources should be preferred in the dietary plans of patients with hepatic encephalopathy because of their better tolerability and their positive impacts on nitrogen balance. Still, with the aim to reduce nitrogen products of the colon, ISHEN suggests to introduce 25–45 g of fiber daily, which is important also to reduce constipation and increase gut motility [155,156].

Moreover, to promote protein synthesis and to improve ammonia detoxification, supplementation with branched-chain amino acid is considered useful by ESPEN 2020 consensus guidelines in cirrhotic subjects that are intolerant to protein intake [150].

In addition, also fat intake should be carefully considered. The percentage of calories from fats should be between 25–30%, since in a cirrhotic liver, metabolism of long-chain fatty acids is impaired and particularly in cases of steatorrhea it is recommended to use foods rich in medium-chain fatty acids (milk and coconut oil) [146].

Finally, in cases of ascites, the energy and protein intake should be calculated relying on dry body weight, approximated to ideal body weight. Importantly, to reduce the activation of renin-angiotensin system, those patients should follow a sodium restricted diet (<2 g per day) [157].

In conclusion, nutrition and metabolic state seem to have an important role in liver disease progression and maintaining the state of health, particularly in PLWH.

## 3. Nutritional Suggestions for Low- and Middle-Income Countries

Nutritional suggestions discussed in this review may be not fully applicable in low- and middle-income countries (LMICs). At first, in this setting, nutritional issues in PLWH include malnutrition, undernutrition, primary food insecurity, unavailability of high-quality food, and food security [158]. In addition, if compared with high-income countries, PLWH usually present with complex opportunistic infectious comorbidities, leading to severely undernourished status [159]. On the contrary, metabolic comorbidities are less frequent at diagnosis, because of the young median age of PLWH in LMICs. Accordingly, principal nutritional concerns for physicians involved in treating PLWH living in LMICs should be directed to differentiate cachexia due to opportunistic infections or HIV-related wasting syndrome from cachexia deriving from malnutrition or undernutrition. Secondly, a proper diet should be suggested, on the basis of food availability and habitus, in order to reach the adequate macro- and micro-nutrient intake.

Importantly, at presentation, undernutrition/malnutrition is often caused by both food unavailability (which can be considered a non-communicable comorbidity) and the inflammation caused by HIV and related opportunistic infection. In practice, this does not matter, as once malnutrition has become established, its management must be integrated into the management plan of the subject. However, it should be noticed that ‘malnutrition’ also reflects abnormalities of nutritional status that are not only evident in terms of wasting of body tissue, but more frequently involve the depletion of vitamins and minerals.

Therefore, resuming the possible approach to the nutritional status of PLWH in LMICs (see Table 5): (i) make a differential diagnosis between nutritional cachexia and HIV-related cachexia; (ii) investigate high-quality food availability and food security; (iii) assess undernutrition and the effects of micro-nutrients deficiency; (iv) assess the macro-nutrients balance, with particular focus on protein intake, that could be undereffective in LMICs.

Interestingly, similarly to high-income countries, nutritional concerns in LMICs have now shifted to the lipodystrophy and metabolic alterations related to drug toxicity where ART is now available. Still, despite the introduction of new ART regimens, with higher rate of virologic success and immunologic recovery, weight loss and severe acute malnutrition remain common, especially among late presenters and those non-adherent to antiretroviral regimens [160]. On the other side, malnutrition and reduced access to adequate or quality foods significantly increase non-adherence to ART, but also malabsorption of drugs [161].

On the basis of these considerations, the pivotal role of proper nutrition is evident also in LMICs; however, general nutritional principles and specific suggestions (also according to comorbidities) does not differ from high-income countries; the main differences lie in economic and social disparities.

In conclusion, physicians should, at first, be aware of underlying conditions of PLWH living in LMICs in order to properly assess how to reach their nutritional needs.

## 4. Conclusions

The improvement of quality of life in PLWH requires multiple interventions and a coordinated approach by infectious diseases specialists and other physicians involved in their care, apart from the bare control of viral replication and immunological status. In this sense, a higher attention to nutrition and metabolic complications, by producing tailored dietary guidelines for PLWH that take in consideration their specific needs, is crucial to make a step closer to the “fourth 90”. In particular, ideal BMI and a diet rich in fruits, vegetables, whole grains, and low in refined sugar and saturated fatty acids, such as the Mediterranean diet, are at the basis of a correct management of main PLWH comorbidities. On the contrary, nutritional requirements differ according to CKD stage and eventually the concomitant presence of diabetes. In conclusion, the nutrition tips provided within this review, could help clinicians in the correct nutrition management of PLWH, setting the nutritional needs of PLWH as a part of primary and secondary prevention strategies of main comorbidities.

## Figures and Tables

**Table 1:** Overview of diagnosis and nutritional management of CVD in PLWH.

**Diagnosis of CVD in PLWH**	
Use score to assess CVD risk in PLWH (Framingham equation/Systematic Coronary Risk Evaluation (SCORE)/data Collection on Adverse Events of Anti-HIV Drugs (DAD) group Repeat the score annually	
**Nutritional management of CVD in PLWH**	
Reduction of body weight and abdominal fat is pivotal Desirable targets: BMI of 20–25 kg/m^2^ Waist circumference <94 cm (men) and <80 cm (women)	
Physical activity: at least 30–60 min of moderate physical activity/day	
Smoking: smoking cessation recommended and reduced exposure to passive cigarette smoke	
Alcohol: Moderate consumption acceptable if triglyceride levels are not elevated	
Dietary approaches: The Mediterranean diet is associated with an almost 30% reduced risk of myocardial infarction, stroke, and cardiovascular mortality	
**FOOD GROUP**	**RECOMMENDATION**
Nuts/seeds	≥3 servings/week
Olive oil	≥4 tbsp/day (around 50 mL)
Fresh fruits/vegetables	≥2–3 servings/day
Legumes	≥3 serving/week
Fish, poultry, dairy products	≥3 serving/week
Whole grain cereals	≥2 serving/week
Wine (red, dry)	≥7 glasses/week
Red and processed meats	<1 serving/day
Sweets	<1 serving/day
The dietary approach to stop hypertension (DASH) diet is another dietary approach that reduces CVD incidence, and, in particular, hypertension. Differently from the Mediterranean diet, it demands a minor use of extra-virgin olive oil. The portfolio diet focuses on consumption of “cholesterol-lowering food” such as nuts, apples, oranges, berries, soluble fibers from oats, barley, psyllium, okra or eggplant, vegetal protein from legumes or soy products, and integration with 2 g plant sterols provided in a plant sterol-enriched margarine	
Carbohydrates: total intake around 45–55%, excessive intake is not recommended (due to its untoward effect on plasma HDL-C and TGs levels)	
Total fat intake: >30% is not recommended but not too low (due to possible vitamin E deficiency, which may advance to a reduction of HDL-C). Less than 10% of total calories should derive from saturated fat.	
Micronutrients: о Dietary sodium: <5 g (90 mmol)/day о Dietary cholesterol: <300 mg/day (especially when plasma cholesterol levels are elevated) о Dietary fibers: between 25–40 g per day (hypocholesterolemic effect)	

Legend: CVD = Cardiovascular disease; PLWH = People Living With HIV; BMI = Body Mass Index.

**Table 2:** Overview of diagnosis and nutritional management of diabetes in PLWH [93].

**Diagnosis of Diabetes in PLWH**
**Diagnostic criteria:** Diabetes: о Fasting plasma glucose >7.0 mmol/L (126 mg/dL) or OGTT 2-h value ≥11.1 mmol/L (200 mg/dL) о HbA1c ≥ 6.5% (≥48 mmol/mol) Impaired glucose tolerance: о Fasting plasma glucose <7 mmol/L (126 mg/dL) and OGTT 2-h value mmol/L (mg/dL) 7.8–11.0 (140–199) о HbA1c 5.7–6.4% (39–47 mmol/mol) (prediabetes) Impaired fasting glucose: о Fasting plasma glucose 5.7–6.9 mmol/L (100–125 mg/dL) and OGTT 2-h value <7.8 mmol/L (140 mg/dL) о HbA1c 5.7–6.4% (39–47 mmol/mol) (prediabetes)
OGTT is recommended in PLWH with fasting blood glucose of 5.7–6.9 mmol/L (100–125 mg/dL)
HbA1c underestimates diabetes in PLWH under antiretroviral therapies (abacavir specifically)
HbA1c is not recommended in cases of hemoglobinopathies, increased erythrocyte turnover, severe liver or kidney dysfunction, patient age > 70, or supplementation with iron, vitamin C and E.
**Nutritional management of diabetes in PLWH**
Energy restriction: daily deficit energy of 600 kcal (with meal plans and portion restriction guidance provided) 1200–1500 kcal/day for women 1500–1800 kcal/day for men
Weight reduction: Achieve 7% weight loss in six months
Carbohydrate reduction (sources should be rich in fiber, such as whole grains, fruits, and vegetables) The optimal amount of protein is about 1–1.5 g/kg body weight (until 20–30%) and 0.8 g per kg of body weight in cases of kidney impairment (microalbuminuria and reduced glomerular filtrate) Fat intake: limit saturated fat (<10% of mean total daily energy intake) and prefer monounsaturated fat (nuts, seeds, olive oil, and fish (in particular salmon, tuna, anchovy, mackerel, herring)
Restrict added sugar to 25 g per day or less Sodium restriction: <6 g salt daily (<2.5 g sodium per day)
Take 10,000 steps per day

Legend: PLWH = People Living With HIV; OGTT: oral glucose tolerance test; HbA1c = glycosylated hemoglobin.

**Table 3:** Overview of diagnosis and nutritional management of CKD in PLWH.

**Diagnosis management of CKD in PLWH**
CKD-EPI is the equation to estimate GFR in PLW
Screen for proteinuria with urine dipstick If urine dipstick is ≥1+, to check UA/C or UP/C to screen for glomerular disease and both glomerular and tubular disease, respectively In cases of tubular proteinuria due to drug nephrotoxicity, UP/C instead of UA/C is the more appropriate marker
**Nutritional management of CKD in PLWH**
In subjects with CKD, the resting energy expenditure is higher if compared to non-CKD (insufficient energy intake could lead to protein catabolism and consequently to a negative nitrogen balance)
Total caloric intake: 25–35 kcal per kg of body weight
Protein restriction with GFR ≤50 mL/minute/1.73 m^2^: о Non-diabetic patients: a low-protein diet providing 0.55–0.60 g dietary protein per kg of body weight per day or a very low-protein diet providing 0.28–0.43 g dietary protein per kg of body weight per day with additional keto acid/amino acid analogs to meet protein requirements о Diabetic patients: protein intake of 0.6–0.8 g per kg of body weight to maintain a stable nutritional status and optimize glycemic control о A patient on maintenance hemodialysis and peritoneal dyalisis without diabetes but metabolically stable and with diabetes: 1.0–1.2 g/kg body weight of proteins
Adjustments of water and electrolyte intake (stage 3–5 of CKD): о Potassium and phosphorus intake to maintain serum levels within normal range о Sodium intake to <2.3 g/die о Total elemental calcium intake of 800–1000 mg/d (including dietary calcium, calcium supplementation and calcium-based phosphate binders) in adults with CKD 3–4 not taking active vitamin D analogs; and a tailored adjustment for CKD stage 5
Mediterranean diet and higher consumption of fruits and vegetables for CKD patients are suggested

Legend: PLWH = People Living With HIV; CKD = Chronic Kidney Disease; UA/C = urine albumin/creatinine; UP/C = urine protein/creatinine; GFR = Glomerular Filtration Rate; CKD-EPI = Chronic Kidney Disease Epidemiology Collaboration.

**Table 4:** Overview of diagnosis and nutritional management of liver diseases in PLWH.

**Diagnosis of liver diseases in PLWH**
NAFLD: transient elastography with controlled attenuation parameter NASH: a biopsy showing steatosis, hepatocyte ballooning, and lobular inflammation is necessary
**Nutritional management of liver disease in PLWH**
In cases of NAFLD or NASH, lifestyle intervention and weight loss are crucial (first approach) Hypocaloric diet (allows the mobilization of fatty acids from liver): о the reduction of at least 3–5% of total bodyweight is sufficient to markedly improve steatosis о the reduction of 7–10% of total bodyweight improves fibrosis Weight loss could produce a reduction of up to 60% of liver triglycerides content, reduction of free fatty acids uptake, and improvement of insulin resistance The Mediterranean diet is recommended as it causes improvement of liver steatosis even in the absence of weight loss
In cases of CIRRHOSIS: Caloric intake should be of 35 to 40 kcal/kg/d Protein intake should be of 1.2 g/kg/d It is necessary to consume multiple meals during the day to avoid hypoglycemia by eating at least 45% to 65% of total caloric intake as carbohydrates (even in case of diabetes) Fats intake should be between 25–30% (it is recommended to use food rich in medium-chain fatty acids, such as milk and coconut oil) Liposoluble vitamins supplementation (A, D, E and K) is important It is suggested introduce to introduce 25–45 g of fiber daily (to reduce constipation and increase gut motility)
In cases of ASCITES: Calories and protein intake should be calculated based on dry body weight Reduce sodium intake (<2 g/die)

Legend: PLWH = People Living With HIV; NAFLD = Non-alcoholic fatty liver disease; NASH = Non-alcoholic Steatohepatitis.

**Table 5:** Overview of diagnosis and nutritional management of PLWH in low- and middle-income countries.

Nutritional suggestions for low- and middle- income countries
Approach to the nutritional status of PLWH in LMICs: make a differential diagnosis between nutritional cachexia and HIV-related cachexia; investigate high quality food availability, food security; assess undernutrition and effects of micro-nutrients deficiency; assess macro-nutrients balance, with particular focus on protein intake, that could be undereffective in LMICs; comorbidities’ nutritional needs do not differ from those of high-income countries even if economic and social disparities make them more difficult to achieve.

Legend: PLWH = People Living With HIV; LMICs = low- and middle-income Countries.

## References

[1] Joint United Nations Programme on HIV/AIDS (2014). 90-90-90 an Ambitious Treatment Target to Help end the AIDS Epidemic. http://www.unaids.org/en/resources/documents/2017/90-90-90.

[2] Harris T.G., Rabkin M., El-Sadr W.M. (2018). Achieving the fourth 90: Healthy aging for people living with HIV. AIDS.

[3] Lazarus J.V., Safreed-Harmon K., Barton S.E., Costagliola D., Dedes N., Del Amo Valero J., Gatell J.M., Baptista-Leite R., Mendão L., Porter K. (2016). Beyond viral suppression of HIV—the new quality of life frontier. BMC Med..

[4] Poliseno M., Bavaro D.F., Di Gennaro F., De Vita G., Girardi E., Saracino A., Monno L., Angarano G., Lo Caputo S. (2020). Lost to follow-up: A challenge over 10 years. AIDS Care.

[5] Willig A.L., Overton E.T. (2016). Metabolic Complications and Glucose Metabolism in HIV Infection: A Review of the Evidence. Curr. HIV/AIDS Rep..

[6] D’Arminio Monforte A., Diaz-Cuervo H., De Luca A., Maggiolo F., Cingolani A., Bonora S., Castagna A., Girardi E., Antinori A., Lo Caputo S. (2019). ICONA Foundation Study Group. Evolution of major non-HIV-related comorbidities in HIV-infected patients in the Italian Cohort of Individuals, Naïve for Antiretrovirals (ICONA) Foundation Study cohort in the period 2004–2014. HIV Med..

[7] Deeks S.G. (2011). HIV infection, inflammation, immunosenescence, and aging. Annu. Rev. Med..

[8] Forman D., Bulwer B.E. (2006). Cardiovascular disease: Optimal approaches to risk factor modification of diet and lifestyle. Curr. Treat Options Cardiovasc. Med..

[9] Waśkiewicz A., Szcześniewska D., Szostak-Węgierek D., Kwaśniewska M., Pająk A., Stepaniak U., Kozakiewicz K., Tykarski A., Zdrojewski T., Zujko M.E. (2016). Are dietary habits of the Polish population consistent with the recommendations for prevention of cardiovascular disease?—WOBASZ II project. Kardiol. Pol..

[10] Mach F., Baigent C., Catapano A.L., Koskinas K.C., Casula M., Badimon L., Chapman M.J., De Backer G.G., Delgado V., Ference B.A. (2020). ESC Scientific Document Group, 2019 ESC/EAS Guidelines for the management of dyslipidaemias: Lipid modification to reduce cardiovascular risk: The Task Force for the management of dyslipidaemias of the European Society of Cardiology (ESC) and European Atherosclerosis Society (EAS). Eur. Heart J..

[11] Ponikowski P., Voors A.A., Anker S.D., Bueno H., Cleland J.G., Coats A.J., Falk V., González-Juanatey J.R., Harjola V.P., Jankowska E.A. (2016). 2016 ESC Guidelines for the diagnosis and treatment of acute and chronic heart failure. Kardiol. Pol..

[12] Shivappa N. (2019). Diet and Chronic Diseases: Is There a Mediating Effect of Inflammation?. Nutrients.

[13] Ávila-Escalante M.L., Coop-Gamas F., Cervantes-Rodríguez M., Méndez-Iturbide D., Aranda-González I.I. (2020). The effect of diet on oxidative stress and metabolic diseases-Clinically controlled trials. J. Food Biochem..

[14] Jiang H., Zhou Y., Tang W. (2020). Maintaining HIV care during the COVID-19 pandemic. Lancet HIV.

[15] Guaraldi G., Milic J., Martinez E., Kamarulzaman A., Mussini C., Waters L., Pozniak A., Mallon P., Rockstroh J., Lazarus J.V. (2020). HIV care models during the COVID-19 era. Clin. Infect. Dis..

[16] Waterfield K.C., Shah G.H., Etheredge G.D., Ikhile O. (2021). Consequences of COVID-19 crisis for persons with HIV: The impact of social determinants of health. BMC Public Health.

[17] Armocida B., Formenti B., Ussai S., Palestra F., Missoni E. (2020). The Italian health system and the COVID-19 challenge. Lancet Public Health.

[18] Senthilingam M. (2021). Covid-19 has made the obesity epidemic worse, but failed to ignite enough action. BMJ.

[19] Kaplan R.C., Hanna D.B., Kizer J.R. (2016). Recent Insights into Cardiovascular Disease (CVD) Risk Among HIV-Infected Adults. Curr. HIV/AIDS Rep..

[20] So-Armah K., Freiberg M.S. (2018). HIV and Cardiovascular Disease: Update on Clinical Events, Special Populations, and Novel Biomarkers. Curr. HIV/AIDS Rep..

[21] Alonso A., Barnes A.E., Guest J.L., Shah A., Shao I.Y., Marconi V. (2019). HIV Infection and Incidence of Cardiovascular Diseases: An Analysis of a Large Healthcare Database. J. Am. Heart Assoc..

[22] Lang S., Mary-Krause M., Simon A., Partisani M., Gilquin J., Cotte L., Boccara F., Costagliola D. (2012). French Hospital Database on HIV (FHDH)–ANRS CO4. HIV replication and immune status are independent predictors of the risk of myocardial infarction in HIV-infected individuals. Clin. Infect. Dis..

[23] D’Ascenzo F., Cerrato E., Appleton D., Moretti C., Calcagno A., Abouzaki N., Vetrovec G., Lhermusier T., Carrie D., Das Neves B. (2014). Percutaneous coronary intervention and surgical revascularization in HIV Database (PHD) Study Investigators. Prognostic indicators for recurrent thrombotic events in HIV-infected patients with acute coronary syndromes: Use of registry data from 12 sites in Europe, South Africa and the United States. Thromb. Res..

[24] Chomont N., El-Far M., Ancuta P., Trautmann L., Procopio F.A., Yassine-Diab B., Boucher G., Boulassel M.R., Ghattas G., Brenchley J.M. (2009). HIV reservoir size and persistence are driven by T cell survival and homeostatic proliferation. Nat. Med..

[25] Chun T.W., Nickle D.C., Justement J.S., Meyers J.H., Roby G., Hallahan C.W., Kottilil S., Moir S., Mican J.M., Mullins J.I. (2008). Persistence of HIV in gut-associated lymphoid tissue despite long-term antiretroviral therapy. J. Infect. Dis..

[26] Lorenzo-Redondo R., Fryer H.R., Bedford T., Kim E.Y., Archer J., Pond S.L.K., Chung Y.S., Penugonda S., Chipman J., Fletcher C.V. (2016). Persistent HIV-1 replication maintains the tissue reservoir during therapy. Nature.

[27] Angelovich T.A., Hearps A.C., Maisa A., Martin G.E., Lichtfuss G.F., Cheng W.-J., Palmer C.S., Landay A.L., Crowe S.M., Jaworowski A. (2015). Viremic and Virologically Suppressed HIV Infection Increases Age-Related Changes to Monocyte Activation Equivalent to 12 and 4 Years of Aging, Respectively. JAIDS J. Acquir. Immune Defic. Syndr..

[28] Karim R., Mack W.J., Kono N., Tien P.C., Anastos K., Lazar J., Young M., Desai S., Golub E.T., Kaplan R.C. (2014). T-cell activation, both pre- and post-HAART levels, correlates with carotid artery stiffness over 6.5 years among HIV-infected women in the WIHS. J. Acquir. Immune Defic. Syndr..

[29] Nordell A.D., McKenna M., Borges Á.H., Duprez D., Neuhaus J., Neaton J.D. (2014). INSIGHT SMART, ESPRIT Study Groups; SILCAAT Scientific Committee. Severity of cardiovascular disease outcomes among patients with HIV is related to markers of inflammation and coagulation. J. Am. Heart Assoc..

[30] Zidar D.A., Juchnowski S., Ferrari B., Clagett B., Pilch-Cooper H.A., Rose S., Rodriguez B., McComsey G.A., Sieg S.F., Mehta N.N. (2015). Oxidized LDL Levels Are Increased in HIV Infection and May Drive Monocyte Activation. J. Acquir. Immune Defic. Syndr..

[31] Kulkarni M.M., Ratcliff A.N., Bhat M., Alwarawrah Y., Hughes P., Arcos J., Loiselle D., Torrelles J.B., Funderburg N.T., Haystead T.A. (2017). Cellular fatty acid synthase is required for late stages of HIV-1 replication. Retrovirology.

[32] Biava P.M., Norbiato G. (2015). Getting an Insight into the Complexity of Major Chronic Inflammatory and Degenerative Diseases: A Potential New Systemic Approach to Their Treatment. Curr. Pharm. Biotechnol..

[33] Ballegaard V., Ralfkiaer U., Pedersen K.K., Hove M., Koplev S., Brændstrup P., Ryder L.P., Madsen H.O., Gerstoft J., Grønbæk K. (2017). MicroRNA-210, MicroRNA-331, and MicroRNA-7 Are Differentially Regulated in Treated HIV-1-Infected Individuals and Are Associated with Markers of Systemic Inflammation. J. Acquir. Immune Defic. Syndr..

[34] Guadalupe M., Reay E., Sankaran S., Prindiville T., Flamm J., McNeil A., Dandekar S. (2003). Severe CD4+ T-cell depletion in gut lymphoid tissue during primary human immunodeficiency virus type 1 infection and substantial delay in restoration following highly active antiretroviral therapy. J. Virol..

[35] Guadalupe M., Sankaran S., George M.D., Reay E., Verhoeven D., Shacklett B.L., Flamm J., Wegelin J., Prindiville T., Dandekar S. (2006). Viral suppression and immune restoration in the gastrointestinal mucosa of human immunodeficiency virus type 1-infected patients initiating therapy during primary or chronic infection. J. Virol..

[36] Mattapallil J.J., Douek D.C., Hill B., Nishimura Y., Martin M., Roederer M. (2005). Massive infection and loss of memory CD4+ T cells in multiple tissues during acute SIV infection. Nature.

[37] Brenchley J.M., Price D.A., Schacker T.W., Asher T.E., Silvestri G., Rao S., Kazzaz Z., Bornstein E., Lambotte O., Altmann D. (2006). Microbial translocation is a cause of systemic immune activation in chronic HIV infection. Nat. Med..

[38] Dinh D.M., Volpe G.E., Duffalo C., Bhalchandra S., Tai A.K., Kane A.V., Wanke C.A., Ward H.D. (2015). Intestinal microbiota, microbial translocation, and systemic inflammation in chronic HIV infection. J. Infect. Dis..

[39] Hunt P.W., Sinclair E., Rodriguez B., Shive C., Clagett B., Funderburg N., Robinson J., Huang Y., Epling L., Martin J.N. (2014). Gut epithelial barrier dysfunction and innate immune activation predict mortality in treated HIV infection. J. Infect. Dis..

[40] Jiang W., Lederman M.M., Hunt P., Sieg S.F., Haley K., Rodriguez B., Landay A., Martin J., Sinclair E., Asher A.I. (2009). Plasma levels of bacterial DNA correlate with immune activation and the magnitude of immune restoration in persons with antiretroviral-treated HIV infection. J. Infect. Dis..

[41] Alvarez A., Orden S., Andújar I., Collado-Diaz V., Núñez-Delgado S., Galindo M.J., Estrada V., Apostolova N., Esplugues J.V. (2017). Cardiovascular toxicity of abacavir: A clinical controversy in need of a pharmacological explanation. AIDS.

[42] De Clercq E. (2016). Tenofovir alafenamide (TAF) as the successor of tenofovir disoproxil fumarate (TDF). Biochem. Pharmacol..

[43] Stellbrink H.J., Lazzarin A., Woolley I., Llibre J.M. (2020). The potential role of bictegravir/emtricitabine/tenofovir alafenamide (BIC/FTC/TAF) single-tablet regimen in the expanding spectrum of fixed-dose combination therapy for HIV. HIV Med..

[44] Friis-Møller N., Weber R., Reiss P., Thiébaut R., Kirk O., d’Arminio Monforte A., Pradier C., Morfeldt L., Mateu S., Law M. (2003). DAD study group. Cardiovascular disease risk factors in HIV patients--association with antiretroviral therapy. Results from the DAD study. AIDS.

[45] Calza L., Manfredi R., Chiodo F. (2003). Hyperlipidaemia in patients with HIV-1 infection receiving highly active antiretroviral therapy: Epidemiology, pathogenesis, clinical course and management. Int. J. Antimicrob. Agents.

[46] Guaraldi G., Stentarelli C., Zona S., Santoro A. (2013). HIV-associated lipodystrophy: Impact of antiretroviral therapy. Drugs.

[47] Greenberg L., Ryom L., Neesgaard B., Wandeler G., Staub T., Gisinger M., Skoll M., Günthard H.F., Scherrer A., Mussini C. (2020). Clinical outcomes of two-drug regimens vs. three-drug regimens in antiretroviral treatment-experienced people living with HIV. Clin. Infect. Dis..

[48] Cento V., Perno C.F. (2020). Two-drug regimens with dolutegravir plus rilpivirine or lamivudine in HIV-1 treatment-naïve, virologically-suppressed patients: Latest evidence from the literature on their efficacy and safety. J. Glob. Antimicrob. Resist..

[49] Piepoli M.F., Hoes A.W., Agewall S., Albus C., Brotons C., Catapano A.L., Cooney M.T., Corrà U., Cosyns B., Deaton C. (2016). 2016 European Guidelines on cardiovascular disease prevention in clinical practice: The Sixth Joint Task Force of the European Society of Cardiology and Other Societies on Cardiovascular Disease Prevention in Clinical Practice (constituted by representatives of 10 societies and by invited experts) Developed with the special contribution of the European Association for Cardiovascular Prevention & Rehabilitation (EACPR). Eur. Heart J..

[50] Ryom L., Cotter A., De Miguel R., Béguelin C., Podlekareva D., Arribas J.R., Marzolini C., Mallon P., Rauch A., Kirk O. (2020). 2019 update of the European AIDS Clinical Society Guidelines for treatment of people living with HIV version 10.0. HIV Med..

[51] Rychter A.M., Ratajczak A.E., Zawada A., Dobrowolska A., Krela-Kaźmierczak I. (2020). Non-Systematic Review of Diet and Nutritional Risk Factors of Cardiovascular Disease in Obesity. Nutrients.

[52] Arnett D.K., Blumenthal R.S., Albert M.A., Buroker A.B., Goldberger Z.D., Hahn E.J., Himmelfarb C.D., Khera A., Lloyd-Jones D., McEvoy J.W. (2019). 2019 ACC/AHA Guideline on the Primary Prevention of Cardiovascular Disease: A Report of the American College of Cardiology/American Heart Association Task Force on Clinical Practice Guidelines. Circulation.

[53] Estruch R., Ros E., Salas-Salvadó J., Covas M.I., Corella D., Arós F., Gómez-Gracia E., Ruiz-Gutiérrez V., Fiol M., Lapetra J. (2018). PREDIMED Study Investigators. Primary Prevention of Cardiovascular Disease with a Mediterranean Diet Supplemented with Extra-Virgin Olive Oil or Nuts. N. Engl. J. Med..

[54] Magnoni M., Scarano P., Vergani V., Berteotti M., Gallone G., Cristell N., Maseri A., Cianflone D. (2020). Impact of adherence to a Mediterranean Diet pattern on patients with first acute myocardial infarction. Nutr. Metab. Cardiovasc. Dis..

[55] Koliaki C., Liatis S., Kokkinos A. (2019). Obesity and cardiovascular disease: Revisiting an old relationship. Metabolism.

[56] Siervo M., Lara J., Chowdhury S., Ashor A., Oggioni C., Mathers J.C. (2015). Effects of the Dietary Approach to Stop Hypertension (DASH) diet on cardiovascular risk factors: A systematic review and meta-analysis. Br. J. Nutr..

[57] Yokoyama Y., Nishimura K., Barnard N.D., Takegami M., Watanabe M., Sekikawa A., Okamura T., Miyamoto Y. (2014). Vegetarian diets and blood pressure: A meta-analysis. JAMA Intern. Med..

[58] Kwok C.S., Umar S., Myint P.K., Mamas M.A., Loke Y.K. (2014). Vegetarian diet, Seventh Day Adventists and risk of cardiovascular mortality: A systematic review and meta-analysis. Int. J. Cardiol..

[59] Chiavaroli L., Nishi S.K., Khan T.A., Braunstein C.R., Glenn A.J., Mejia S.B., Rahelić D., Kahleová H., Salas-Salvadó J., Jenkins D.J.A. (2018). Portfolio Dietary Pattern and Cardiovascular Disease: A Systematic Review and Meta-analysis of Controlled Trials. Prog. Cardiovasc. Dis..

[60] Jenkins D.J., Kendall C.W., Faulkner D., Vidgen E., Trautwein E.A., Parker T.L., Marchie A., Koumbridis G., Lapsley K.G., Josse R.G. (2002). A dietary portfolio approach to cholesterol reduction: Combined effects of plant sterols, vegetable proteins, and viscous fibers in hypercholesterolemia. Metabolism.

[61] Hu T., Bazzano L.A. (2014). The low-carbohydrate diet and cardiovascular risk factors: Evidence from epidemiologic studies. Nutr. Metab. Cardiovasc. Dis..

[62] Torres N., Guevara-Cruz M., Velázquez-Villegas L.A., Tovar A.R. (2015). Nutrition and Atherosclerosis. Arch. Med. Res..

[63] Alexander D.D., Miller P.E., Vargas A.J., Weed D.L., Cohen S.S. (2016). Meta-analysis of Egg Consumption and Risk of Coronary Heart Disease and Stroke. J. Am. Coll. Nutr..

[64] Callejo M., Barberá J.A., Duarte J., Perez-Vizcaino F. (2020). Impact of Nutrition on Pulmonary Arterial Hypertension. Nutrients.

[65] Stradling C., Thomas G.N., Hemming K., Taheri S., Taylor S., Ross J., Das S. The Mediterranean portfolio diet in HIV dyslipidaemia: A randomized controlled trial. Proceedings of the Conference on Retroviruses and Opportunistic Infections.

[66] Brown T.T., Cole S.R., Li X., Kingsley L.A., Palella F.J., Riddler S.A., Visscher B.R., Margolick J.B., Dobs A.S. (2005). Antiretroviral therapy and the prevalence and incidence of diabetes mellitus in the multicenter AIDS cohort study. Arch. Intern. Med..

[67] Coelho A.R., Moreira F.A., Santos A.C., Silva-Pinto A., Sarmento A., Carvalho D., Freitas P. (2018). Diabetes mellitus in HIV-infected patients: Fasting glucose, A1c, or oral glucose tolerance test—Which method to choose for the diagnosis?. BMC Infect. Dis..

[68] Brown T.T., Tassiopoulos K., Bosch R.J., Shikuma C., McComsey G.A. (2010). Association between systemic inflammation and incident diabetes in HIV-infected patients after initiation of antiretroviral therapy. Diabetes Care..

[69] Gianotti N., Visco F., Galli L., Barda B., Piatti P., Salpietro S., Bigoloni A., Vinci C., Nozza S., Gallotta G. (2011). Detecting impaired glucose tolerance or type 2 diabetes mellitus by means of an oral glucose tolerance test in HIV-infected patients. HIV Med..

[70] Galli L., Salpietro S., Pellicciotta G., Galliani A., Piatti P., Hasson H., Guffanti M., Gianotti N., Bigoloni A., Lazzarin A. (2012). Risk of type 2 diabetes among HIV-infected and healthy subjects in Italy. Eur. J. Epidemiol..

[71] Bavaro D.F., Di Carlo D., Rossetti B., Bruzzone B., Vicenti I., Pontali E., Zoncada A., Lombardi F., Di Giambenedetto S., Borghi V. (2020). Pretreatment HIV drug resistance and treatment failure in non-Italian HIV-1-infected patients enrolled in ARCA. Antivir. Ther..

[72] Grunfeld C. (2008). Insulin resistance in HIV infection: Drugs, host responses, or restoration to health?. Top HIV Med..

[73] Brown T.T., Li X., Cole S.R., Kingsley L.A., Palella F.J., Riddler S.A., Chmiel J.S., Visscher B.R., Margolick J.B., Dobs A.S. (2005). Cumulative exposure to nucleoside analogue reverse transcriptase inhibitors is associated with insulin resistance markers in the Multicenter AIDS Cohort Study. AIDS.

[74] Hresko R.C., Hruz P.W. (2011). HIV protease inhibitors act as competitive inhibitors of the cytoplasmic glucose binding site of GLUTs with differing affinities for GLUT1 and GLUT4. PLoS ONE.

[75] Yarasheski K.E., Tebas P., Sigmund C., Dagogo-Jack S., Bohrer A., Turk J., Halban P.A., Cryer P.E., Powderly W.G. (1999). Insulin resistance in HIV protease inhibitor-associated diabetes. J. Acquir. Immune. Defic. Syndr..

[76] Trabattoni D., Schenal M., Cesari M., Castelletti E., Pacei M., Goldberg B., Gori A., Clerici M. (2006). Low interleukin-10 production is associated with diabetes in HIV-infected patients undergoing antiviral therapy. Med. Microbiol. Immunol..

[77] Nolan N.S., Adamson S., Reeds D., O’Halloran J.A. (2021). Bictegravir-Based Antiretroviral Therapy-Associated Accelerated Hyperglycemia and Diabetes Mellitus. Open Forum Infect. Dis..

[78] Shah S., Hill A. (2021). Risks of metabolic syndrome and diabetes with integrase inhibitor-based therapy. Curr. Opin. Infect. Dis..

[79] Van Wyk J., Ajana F., Bisshop F., De Wit S., Osiyemi O., Portilla Sogorb J., Routy J.P., Wyen C., Ait-Khaled M., Nascimento M.C. (2020). Efficacy and Safety of Switching to Dolutegravir/Lamivudine Fixed-Dose 2-Drug Regimen vs Continuing a Tenofovir Alafenamide-Based 3- or 4-Drug Regimen for Maintenance of Virologic Suppression in Adults Living With Human Immunodeficiency Virus Type 1: Phase 3, Randomized, Noninferiority TANGO Study. Clin. Infect. Dis..

[80] Zheng Y., Ley S.H., Hu F.B. (2018). Global aetiology and epidemiology of type 2 diabetes mellitus and its complications. Nat. Rev. Endocrinol..

[81] Duncan A.D., Peters B.S., Rivas C., Goff L.M. (2020). Reducing risk of Type 2 diabetes in HIV: A mixed-methods investigation of the STOP-Diabetes diet and physical activity intervention. Diabet Med..

[82] Kahn S.E., Hull R.L., Utzschneider K.M. (2006). Mechanisms linking obesity to insulin resistance and type 2 diabetes. Nature.

[83] Rising R., Harper I.T., Fontvielle A.M., Ferraro R.T., Spraul M., Ravussin E. (1994). Determinants of total daily energy expenditure: Variability in physical activity. Am. J. Clin. Nutr..

[84] Ge L., Sadeghirad B., Ball G.D.C., da Costa B.R., Hitchcock C.L., Svendrovski A., Kiflen R., Quadri K., Kwon H.Y., Karamouzian M. (2020). Comparison of dietary macronutrient patterns of 14 popular named dietary programmes for weight and cardiovascular risk factor reduction in adults: Systematic review and network meta-analysis of randomised trials. BMJ.

[85] American Diabetes Association (2019). 5. Lifestyle Management: Standards of Medical Care in Diabetes-2019. Diabetes Care.

[86] Ghazzawi H., Mustafa S. (2019). Effect of High-Protein Breakfast Meal on Within-DayAppetite Hormones Cholecystokinin, Ghrelin, Peptide Yy, Glucagone Like Peptide-1 in adults. Clin. Nutr. Exp..

[87] Hoel H., Hove-Skovsgaard M., Hov J.R., Gaardbo J.C., Holm K., Kummen M., Rudi K., Nwosu F., Valeur J., Gelpi M. (2018). Impact of HIV and Type 2 diabetes on Gut Microbiota Diversity, Tryptophan Catabolism and Endothelial Dysfunction. Sci. Rep..

[88] Cunningham-Rundles S., Ahrné S., Johann-Liang R., Abuav R., Dunn-Navarra A.M., Grassey C., Bengmark S., Cervia J.S. (2011). Effect of probiotic bacteria on microbial host defense, growth, and immune function in human immunodeficiency virus type-1 infection. Nutrients.

[89] Yang O.O., Kelesidis T., Cordova R., Khanlou H. (2014). Immunomodulation of antiretroviral drug-suppressed chronic HIV-1 infection in an oral probiotic double-blind placebo-controlled trial. AIDS Res. Hum. Retrovir..

[90] Connolly N.C., Riddler S.A., Rinaldo C.R. (2005). Proinflammatory cytokines in HIV disease-a review and rationale for new therapeutic approaches. AIDS Rev..

[91] Merlini E., Bai F., Bellistrì G.M., Tincati C., d’Arminio Monforte A., Marchetti G. (2011). Evidence for polymicrobic flora translocating in peripheral blood of HIV-infected patients with poor immune response to antiretroviral therapy. PLoS ONE.

[92] Gad M., Ravn P., Søborg D.A., Lund-Jensen K., Ouwehand A.C., Jensen S.S. (2011). Regulation of the IL-10/IL-12 axis in human dendritic cells with probiotic bacteria. FEMS Immunol. Med. Microbiol..

[93] (2020). EACS Guidelines Version 10.1. https://www.eacsociety.org/media/guidelines-10.1_30032021_1.pdf.

[94] Ando M., Yanagisawa N. (2015). Epidemiology, clinical characteristics, and management of chronic kidney disease in human immunodeficiency virus-infected patients. World J. Nephrol..

[95] Mocroft A., Neuhaus J., Peters L., Ryom L., Bickel M., Grint D., Koirala J., Szymczak A., Lundgren J., Ross M.J. (2012). Hepatitis B and C co-infection are independent predictors of progressive kidney disease in HIV-positive, antiretroviral-treated adults. PLoS ONE.

[96] Ryom L., Mocroft A., Kirk O., Ross M., Reiss P., Fux C.A., Morlat P., Moranne O., Smith C., El-Sadr W. (2014). Predictors of advanced chronic kidney disease and end-stage renal disease in HIV-positive persons. AIDS.

[97] George E., Lucas G.M., Nadkarni G.N., Fine D.M., Moore R., Atta M.G. (2010). Kidney function and the risk of cardiovascular events in HIV-1-infected patients. AIDS.

[98] Mocroft A., Ryom L., Begovac J., Monforte A.D., Vassilenko A., Gatell J., Florence E., Ormaasen V., Kirk O., Lundgren J.D. (2014). Deteriorating renal function and clinical outcomes in HIV-positive persons. AIDS.

[99] Mallipattu S.K., Salem F., Wyatt C.M. (2014). The changing epidemiology of HIV-related chronic kidney disease in the era of antiretroviral therapy. Kidney Int..

[100] Petersen N., Knudsen A.D., Mocroft A., Kirkegaard-Klitbo D., Arici E., Lundgren J., Benfield T., Oturai P., Nordestgaard B.G., Feldt-Rasmussen B. (2019). Prevalence of impaired renal function in virologically suppressed people living with HIV compared with controls: The Copenhagen Comorbidity in HIV Infection (COCOMO) study. HIV Med..

[101] Bertoldi A., De Crignis E., Miserocchi A., Bon I., Musumeci G., Longo S., D’Urbano V., La Manna G., Calza L., Re M.C. (2017). HIV and kidney: A dangerous liaison. New Microbiol..

[102] Faulhaber J.R., Nelson P.J. (2007). Virus-induced cellular immune mechanisms of injury to the kidney. Clin. J. Am. Soc. Nephrol..

[103] Ostrowski S.R., Katzenstein T.L., Pedersen B.K., Gerstoft J., Ullum H. (2008). Residual viraemia in HIV-1-infected patients with plasma viral load. Scand J. Immunol..

[104] Bastard J.P., Soulié C., Fellahi S., Haïm-Boukobza S., Simon A., Katlama C., Calvez V., Marcelin A.G., Capeau J. (2012). Circulating interleukin-6 levels correlate with residual HIV viraemia and markers of immune dysfunction in treatment-controlled HIV-infected patients. Antivir. Ther..

[105] Snyder A., Alsauskas Z.C., Leventhal J.S., Rosenstiel P.E., Gong P., Chan J.J., Barley K., He J.C., Klotman M.E., Ross M.J. (2010). HIV-1 viral protein r induces ERK and caspase-8-dependent apoptosis in renal tubular epithelial cells. AIDS.

[106] Selvaraj S., Ghebremichael M., Li M., Foli Y., Langs-Barlow A., Ogbuagu A., Barakat L., Tubridy E., Edifor R., Lam W. (2014). Antiretroviral therapy-induced mitochondrial toxicity: Potential mechanisms beyond polymerase-γ inhibition. Clin. Pharmacol. Ther..

[107] Karras A., Lafaurie M., Furco A., Bourgarit A., Droz D., Sereni D., Legendre C., Martinez F., Molina J.M. (2003). Tenofovir-related nephrotoxicity in human immunodeficiency virus-infected patients: Three cases of renal failure, Fanconi syndrome, and nephrogenic diabetes insipidus. Clin. Infect. Dis..

[108] Hsu R., Brunet L., Fusco J., Beyer A., Prajapati G., Wyatt C., Wohlfeiler M., Fusco G. (2021). Risk of chronic kidney disease in people living with HIV by tenofovir disoproxil fumarate (TDF) use and baseline D:A:D chronic kidney disease risk score. HIV Med..

[109] Brewster U.C., Perazella M.A. (2004). Acute interstitial nephritis associated with atazanavir, a new protease inhibitor. Am. J. Kidney Dis..

[110] Izzedine H., M’rad M.B., Bardier A., Daudon M., Salmon D. (2007). Atazanavir crystal nephropathy. AIDS.

[111] Anderson P.L., Lichtenstein K.A., Gerig N.E., Kiser J.J., Bushman L.R. (2007). Atazanavir-containing renal calculi in an HIV-infected patient. AIDS.

[112] Mocroft A., Kirk O., Reiss P., De Wit S., Sedlacek D., Beniowski M., Gatell J., Phillips A.N., Ledergerber B., Lundgren J.D. (2010). Estimated glomerular filtration rate, chronic kidney disease and antiretroviral drug use in HIV-positive patients. AIDS.

[113] Hepatitis B. (2017). EASL Guidelines, 2019; Preexposure Prophylaxis for the Prevention of HIV Infection in the United States—2017 Update Clinical Practice Guideline. J. Hepatol..

[114] Chazot R., Botelho-Nevers E., Frésard A., Maillard N., Mariat C., Lucht F., Gagneux-Brunon A. (2017). Diagnostic challenges of kidney diseases in HIV-infected patients. Expert Rev. Anti. Infect. Ther..

[115] Neyra R., Chen K.Y., Sun M., Shyr Y., Hakim R.M., Ikizler T.A. (2003). Increased resting energy expenditure in patients with end-stage renal disease. JPEN J. Parenter Enteral Nutr..

[116] Ikizler T.A., Burrowes J.D., Byham-Gray L.D., Campbell K.L., Carrero J.J., Chan W., Fouque D., Friedman A.N., Ghaddar S., Goldstein-Fuchs D.J. (2020). KDOQI Clinical Practice Guideline for Nutrition in CKD: 2020 Update. Am. J. Kidney Dis..

[117] Yan B., Su X., Xu B., Qiao X., Wang L. (2018). Effect of diet protein restriction on progression of chronic kidney disease: A systematic review and meta-analysis. PLoS ONE.

[118] Zha Y., Qian Q. (2017). Protein Nutrition and Malnutrition in CKD and ESRD. Nutrients.

[119] Fantuzzi A.L., Lugli F., Giannini R. (2014). The opinion of patients with chronic renal disease on low-protein foods. G. Tec. Nefrol. Dial..

[120] D’Alessandro C., Rossi A., Innocenti M., Ricchiuti G., Bozzoli L., Sbragia G., Meola M., Cupisti A. (2013). Dietary protein restriction for renal patients: Don’t forget protein-free foods. J. Ren. Nutr..

[121] Bradbury B.D., Fissell R.B., Albert J.M., Anthony M.S., Critchlow C.W., Pisoni R.L., Port F.K., Gillespie B.W. (2007). Predictors of early mortality among incident US hemodialysis patients in the Dialysis Outcomes and Practice Patterns Study (DOPPS). Clin. J. Am. Soc. Nephrol..

[122] Adeney K.L., Siscovick D.S., Ix J.H., Seliger S.L., Shlipak M.G., Jenny N.S., Kestenbaum B.R. (2009). Association of serum phosphate with vascular and valvular calcification in moderate CKD. J. Am. Soc. Nephrol..

[123] Reaich D., Channon S.M., Scrimgeour C.M., Daley S.E., Wilkinson R., Goodship T.H. (1993). Correction of acidosis in humans with CRF decreases protein degradation and amino acid oxidation. Am. J. Physiol..

[124] Montemurno E., Cosola C., Dalfino G., Daidone G., De Angelis M., Gobbetti M., Gesualdo L. (2014). What would you like to eat, Mr CKD Microbiota? A Mediterranean Diet, please!. Kidney Blood Press Res..

[125] Ioannou G.N., Bryson C.L., Weiss N.S., Miller R., Scott J.D., Boyko E.J. (2013). The prevalence of cirrhosis and hepatocellular carcinoma in patients with human immunodeficiency virus infection. Hepatology.

[126] Thio C.L., Seaberg E.C., Skolasky R., Phair J., Visscher B., Muñoz A., Thomas D.L. (2002). Multicenter AIDS Cohort Study. HIV-1, hepatitis B virus, and risk of liver-related mortality in the Multicenter Cohort Study (MACS). Lancet.

[127] Bavaro D.F., Saracino A., Fiordelisi D., Bruno G., Ladisa N., Monno L., Angarano G. (2019). Influence of HLA-B18 on liver fibrosis progression in a cohort of HIV/HCV coinfected individuals. J. Med. Virol..

[128] Lin W., Weinberg E.M., Tai A.W., Peng L.F., Brockman M.A., Kim K.A., Kim S.S., Borges C.B., Shao R.X., Chung R.T. (2008). HIV increases HCV replication in a TGF-beta1-dependent manner. Gastroenterology.

[129] Hong F., Tuyama A., Lee T.F., Loke J., Agarwal R., Cheng X., Garg A., Fiel M.I., Schwartz M., Walewski J. (2009). Hepatic stellate cells express functional CXCR4: Role in stromal cell-derived factor-1alpha-mediated stellate cell activation. Hepatology.

[130] Balagopal A., Ray S.C., De Oca R.M., Sutcliffe C.G., Vivekanandan P., Higgins Y., Mehta S.H., Moore R.D., Sulkowski M.S., Thomas D.L. (2009). Kupffer cells are depleted with HIV immunodeficiency and partially recovered with antiretroviral immune reconstitution. AIDS.

[131] Balagopal A., Philp F.H., Astemborski J., Block T.M., Mehta A., Long R., Kirk G.D., Mehta S.H., Cox A.L., Thomas D.L. (2008). Human immunodeficiency virus-related microbial translocation and progression of hepatitis C. Gastroenterology.

[132] Woreta T.A., Sutcliffe C.G., Mehta S.H., Brown T.T., Higgins Y., Thomas D.L., Torbenson M.S., Moore R.D., Sulkowski M.S. (2011). Incidence and risk factors for steatosis progression in adults coinfected with HIV and hepatitis C virus. Gastroenterology.

[133] Norwood J., Turner M., Bofill C., Rebeiro P., Shepherd B., Bebawy S., Hulgan T., Raffanti S., Haas D.W., Sterling T.R. (2017). Brief Report: Weight Gain in Persons with HIV Switched From Efavirenz-Based to Integrase Strand Transfer Inhibitor-Based Regimens. J. Acquir. Immune Defic. Syndr..

[134] Menard A., Meddeb L., Tissot-Dupont H., Ravaux I., Dhiver C., Mokhtari S., Tomei C., Brouqui P., Colson P., Stein A. (2017). Dolutegravir and weight gain: An unexpected bothering side effect?. AIDS.

[135] Vodkin I., Valasek M.A., Bettencourt R., Cachay E., Loomba R. (2015). Clinical, biochemical and histological differences between HIV-associated NAFLD and primary NAFLD: A case-control study. Aliment Pharmacol. Ther..

[136] Fracanzani A.L., Valenti L., Bugianesi E., Andreoletti M., Colli A., Vanni E., Bertelli C., Fatta E., Bignamini D., Marchesini G. (2008). Risk of severe liver disease in nonalcoholic fatty liver disease with normal aminotransferase levels: A role for insulin resistance and diabetes. Hepatology.

[137] Ratziu V., Giral P., Munteanu M., Messous D., Mercadier A., Bernard M., Morra R., Imbert-Bismut F., Bruckert E., Poynard T. (2007). Screening for liver disease using non-invasive biomarkers (FibroTest, SteatoTest and NashTest) in patients with hyperlipidaemia. Aliment Pharmacol. Ther..

[138] Chen J., Talwalkar J.A., Yin M., Glaser K.J., Sanderson S.O., Ehman R.L. (2011). Early detection of nonalcoholic steatohepatitis in patients with nonalcoholic fatty liver disease by using MR elastography. Radiology.

[139] Wong V.W., Vergniol J., Wong G.L., Foucher J., Chan H.L., Le Bail B., Choi P.C., Kowo M., Chan A.W., Merrouche W. (2010). Diagnosis of fibrosis and cirrhosis using liver stiffness measurement in nonalcoholic fatty liver disease. Hepatology.

[140] Plauth M., Bernal W., Dasarathy S., Merli M., Plank L.D., Schütz T., Bischoff S.C. (2019). ESPEN guideline on clinical nutrition in liver disease. Clin. Nutr..

[141] Chalasani N., Younossi Z., Lavine J.E., Charlton M., Cusi K., Rinella M., Harrison S.A., Brunt E.M., Sanyal A.J. (2018). The diagnosis and management of nonalcoholic fatty liver disease: Practice guidance from the American Association for the Study of Liver Diseases. Hepatology.

[142] Viljanen A.P., Iozzo P., Borra R., Kankaanpää M., Karmi A., Lautamäki R., Järvisalo M., Parkkola R., Rönnemaa T., Guiducci L. (2009). Effect of weight loss on liver free fatty acid uptake and hepatic insulin resistance. J. Clin. Endocrinol. Metab..

[143] Zelber-Sagi S., Salomone F., Mlynarsky L. (2017). The Mediterranean dietary pattern as the diet of choice for non-alcoholic fatty liver disease: Evidence and plausible mechanisms. Liver Int..

[144] Koopman K.E., Caan M.W., Nederveen A.J., Pels A., Ackermans M.T., Fliers E., la Fleur S.E., Serlie M.J. (2014). Hypercaloric diets with increased meal frequency, but not meal size, increase intrahepatic triglycerides: A randomized controlled trial. Hepatology.

[145] Juakiem W., Torres D.M., Harrison S.A. (2014). Nutrition in cirrhosis and chronic liver disease. Clin. Liver Dis..

[146] Cheung K., Lee S.S., Raman M. (2012). Prevalence and mechanisms of malnutrition in patients with advanced liver disease, and nutrition management strategies. Clin. Gastroenterol. Hepatol..

[147] Dudrick S.J., Kavic S.M. (2002). Hepatobiliary nutrition: History and future. J. Hepatobiliary Pancreat. Surg..

[148] Vlahcevic Z.R., Buhac I., Farrar J.T., Bell C.C., Swell L. (1971). Bile acid metabolism in patients with cirrhosis. I. Kinetic aspects of cholic acid metabolism. Gastroenterology.

[149] Tsiaousi E.T., Hatzitolios A.I., Trygonis S.K., Savopoulos C.G. (2008). Malnutrition in end stage liver disease: Recommendations and nutritional support. J. Gastroenterol. Hepatol..

[150] Dasarathy S. (2012). Consilience in sarcopenia of cirrhosis. J. Cachexia Sarcopenia Muscle..

[151] Englesbe M.J., Patel S.P., He K., Lynch R.J., Schaubel D.E., Harbaugh C., Holcombe S.A., Wang S.C., Segev D.L., Sonnenday C.J. (2010). Sarcopenia and mortality after liver transplantation. J. Am. Coll. Surg..

[152] Nakaya Okita K., Suzuki K., Moriwaki H., Kato A., Miwa Y., Shiraishi K., Okuda H., Onji M., Kanazawa H., Tsubouchi H. (2007). Hepatic Nutritional Therapy (HNT) Study Group. BCAA-enriched snack improves nutritional state of cirrhosis. Nutrition.

[153] Plank L.D., Gane E.J., Peng S., Muthu C., Mathur S., Gillanders L., McIlroy K., Donaghy A.J., McCall J.L. (2008). Nocturnal nutritional supplementation improves total body protein status of patients with liver cirrhosis: A randomized 12-month trial. Hepatology.

[154] Córdoba J., López-Hellín J., Planas M., Sabín P., Sanpedro F., Castro F., Esteban R., Guardia J. (2004). Normal protein diet for episodic hepatic encephalopathy: Results of a randomized study. J. Hepatol..

[155] Amodio P., Bemeur C., Butterworth R., Cordoba J., Kato A., Montagnese S., Uribe M., Vilstrup H., Morgan M.Y. (2013). The nutritional management of hepatic encephalopathy in patients with cirrhosis: International Society for Hepatic Encephalopathy and Nitrogen Metabolism Consensus. Hepatology.

[156] Chadalavada R., Sappati Biyyani R.S., Maxwell J., Mullen K. (2010). Nutrition in hepatic encephalopathy. Nutr. Clin. Pract..

[157] Salerno F., Guevara M., Bernardi M., Moreau R., Wong F., Angeli P., Garcia-Tsao G., Lee S.S. (2010). Refractory ascites: Pathogenesis, definition and therapy of a severe complication in patients with cirrhosis. Liver Int..

[158] Gebremichael D.Y., Hadush K.T., Kebede E.M., Zegeye R.T. (2018). Food Insecurity, Nutritional Status, and Factors Associated with Malnutrition among People Living with HIV/AIDS Attending Antiretroviral Therapy at Public Health Facilities in West Shewa Zone, Central Ethiopia. Biomed. Res. Int..

[159] Kelly P., Saloojee H., Chen J.Y., Chung R.T. (2014). Noncommunicable diseases in HIV infection in low- and middle-income countries: Gastrointestinal, hepatic, and nutritional aspects. J. Acquir. Immune Defic. Syndr..

[160] Wanke C.A., Silva M., Knox T.A., Forrester J., Speigelman D., Gorbach S.L. (2000). Weight loss and wasting remain common complications in individuals infected with human immunodeficiency virus in the era of highly active antiretroviral therapy. Clin. Infect. Dis..

[161] Berhe N., Tegabu D., Alemayehu M. (2013). Effect of nutritional factors on adherence to antiretroviral therapy among HIV-infected adults: A case control study in Northern Ethiopia. BMC Infect. Dis..

